# Editorial: Neuroglycobiology

**DOI:** 10.3389/fnmol.2026.1814339

**Published:** 2026-03-18

**Authors:** Cyril Hanus, Robert G. Mealer, Liqin Zhao, Thomas S. Klarić

**Affiliations:** 1 Institute of Psychiatry and Neurosciences of Paris, Paris-Cité University, INSERM UMR 1266, Paris, France; 2 GHU Psychiatry and Neurosciences, Paris, France; 3 Department of Psychiatry, School of Medicine, University of North Carolina at Chapel Hill, Chapel Hill, NC, United States; 4 Department of Genetics, School of Medicine, University of North Carolina at Chapel Hill, Chapel Hill, NC, United States; 5 UNC Neuroscience Center, School of Medicine, University of North Carolina at Chapel Hill, Chapel Hill, NC, United States; 6 Department of Pharmacology and Toxicology, Neuroscience Graduate Program, University of Kansas, Lawrence, KS, United States; 7 Genos Glycoscience Research Laboratory, Zagreb, Croatia

**Keywords:** apolipoprotein E, extracellular matrix, glycosylation, human milk oligosaccharides, Neuroplastin 65, O-mannosylation, Sanfilippo disease, sialylation

**Figure 1:**
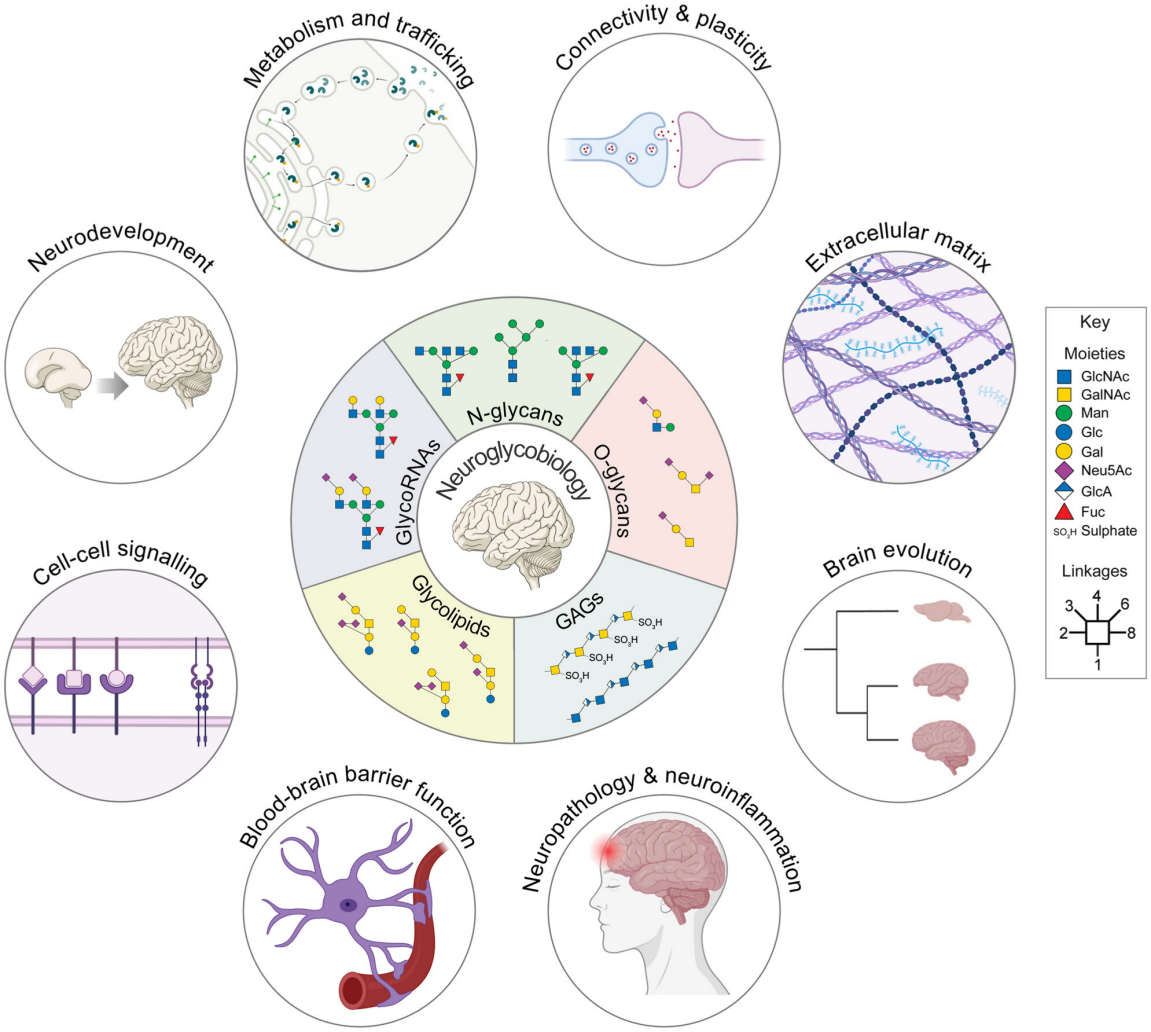
Carbohydrates play a significant role in many fundamental neurobiological processes. Neuroglycobiology is the study of glycoconjugates and their carbohydrates in the nervous system. The inner wheel depicts the major classes of glycans along with some of the most abundant structures found in the brain from each category. Not only does glycosylation increase the complexity and structural diversity of the macromolecules it modifies (i.e., proteins, lipids, and nucleic acids), it also modulates their functions, thus positioning glycans as central regulators of nervous system biology. The icons forming the outer circle illustrate just some of the many neurobiological processes in which glycoconjugates play a central role. Importantly, glycosylation is not a template-driven process encoded by the genome. Instead, it integrates both intrinsic and environmental cues, which means that the glycome adds a dynamic and malleable layer of information to the molecules it modifies. Fuc, fucose; GAGs, glycosaminoglycans; Gal, galactose; GalNAc, *N*-acetylgalactosamine; Glc, glucose; GlcA, glucuronic acid; GlcNAc, *N*-acetylglucosamine; Man, mannose; Neu5Ac, *N*-acetylneuraminic acid; N-glycans, N-linked glycans; O-glycans, O-linked glycans.

Neuroglycobiology sits at the intersection of neuroscience and glycobiology, focusing on how glycans and glycoconjugates shape brain development, function, and disease. Glycosylation influences virtually every level of nervous system biology, from cell–cell communication and extracellular matrix (ECM) organization to synaptic plasticity, myelination, and neuroinflammation (Figure 1). Despite this centrality, brain glycans (and indeed glycans in general) have historically been underexplored, largely due to their structural complexity and the technical challenges associated with their analysis. Methodological advances and a growing recognition of the brain's unique glycosylation landscape have stimulated research activity in the field in recent years. The present Research Topic brings together six articles that highlight the breadth of contemporary neuroglycobiology, spanning human disease, brain development, ECM biology, and protein-specific glycosylation. Collectively, these contributions illustrate how diverse classes of glycans—from glycosaminoglycans to N- and O-linked glycans—contribute to molecular signaling pathways in the healthy and diseased nervous system.

Work by Taherzadeh et al. on Sanfilippo disease, a lysosomal storage disease characterized by buildup of heparan sulfate, neuronal dysfunction, and early death, used a mouse model and human brain samples to investigate previously unexplored white matter changes. In both models, considerable demyelination and axon degeneration were observed using multiple complementary techniques, suggesting that these alterations could serve to monitor disease progression. These findings provide insight into how disrupted glycosaminoglycan clearance may contribute to white matter changes in addition to established gray matter abnormalities, highlighting the complex interplay between carbohydrate classes and neurological diseases.

Golden et al. describe the tolerability of human milk oligosaccharide supplementation in healthy newborns. Specifically, 3-sialyllactose and 6 sialyllactose, which contain critical monosaccharides essential for the synthesis of complex brain glycans, including glycolipids and glycoproteins, were administered at fixed doses up to 2 months. Both treatments were well-tolerated, with no impact on animal growth or sialic acid concentrations in the brain. These null results may inform how and when to supplement human milk oligosaccharides, which are present at lower levels in formula and cow's milk than in human milk. Further, these findings could guide the development of therapies for congenital disorders of glycosylation (Park and Marquardt, 2021), where the brain appears uniquely vulnerable to glycosylation deficiencies compared to other organs (Paprocka et al., 2021).

Using ECM generated from genetically edited and decellularized fibroblast cultures, Sharma et al. describe that specific binding domains of fibronectin (FN)—a key organizing component of the ECM formed during wound repair—are important to create an environment more permissive to neuronal growth. Specifically, they show that the III-13 module of the FN heparin II domain, which is responsible for FN interaction with ECM glyco-amino-glycans, tenascin-C and growth factors, regulates ECM composition and favors the growth and maintenance of the axons and dendrites of young cortical neurons in cultures. This study hence provides novel insights on the potential impact of the ECM on brain regeneration after stress and trauma.

Focusing on O-mannosyl glycans, Jiang et al. investigated the expression and distribution of POMGNT1, a key O-glycan modifying enzyme linked to muscle-eye-brain (MEB) disease, in the adult mouse brain. Using western blotting, RT-qPCR, and immunolabeling techniques, they demonstrated that POMGNT1 is widely expressed across various brain regions—most notably in the cerebral cortex and hippocampus. The study further shows that POMGNT1 is predominantly localized in glutamatergic neurons and enriched in the Golgi apparatus, providing cellular insights into the neurophysiological function of this protein.

Neuroplastin 65 (NP65) is a brain-specific glycoprotein and has been shown to play crucial roles in synaptic plasticity, emotional regulation, and cognitive functions. Human NP65 protein contains six potential *N*-glycosylation sites and one *O*-glycosylation site, which contributes 25 kDa, nearly 40%, to the molecular weight of the protein. The study authored by Wu et al. demonstrated that loss of NP65 reduced amyloid plaque formation and alleviated cognitive deficits in the transgenic amyloid precursor protein (APP)/presenilin 1 (PS1) mouse model of Alzheimer's disease, suggesting that NP65 may be involved in pathogenic processes in Alzheimer's disease. Future research will be needed to investigate how glycosylation in NP65 affects its interaction with amyloid beta and synaptic proteins, as well as how altered glycosylation in NP65 contributes to disease onset and progression in Alzheimer's disease and beyond.

Human apolipoprotein E (ApoE) exists in three major variants, ApoE2, ApoE3, and ApoE4, which play distinct roles in the etiology of diseases such as sporadic Alzheimer's disease. The review article, authored by Moon et al., provides a comprehensive summary of the current state of knowledge regarding ApoE glycosylation and sialylation, from their chemistry to biological impact on health and disease. Human ApoE proteins, particularly expressed in the brain, are heavily glycosylated and sialylated, with eight exclusive *O*-linked glycosylation sites experimentally confirmed by at least two independent studies. The species-, tissue-, and variant-specific differences in ApoE glycosylation and sialylation underscore their potentially critical roles in regulating ApoE function within specific cellular environments. The authors call for increased efforts in future studies to advance this important yet largely overlooked frontier of research in the ApoE field.

Together, the articles in this Research Topic underscore the multifaceted roles of glycans in neuronal physiology and pathology, revealing how alterations in glycan metabolism, structure, or localization can have profound consequences for brain function. From white matter pathology in lysosomal storage disease and the neurodevelopmental implications of milk-derived oligosaccharides, to ECM–mediated dendritic outgrowth and glycoproteins implicated in Alzheimer's disease pathology, these studies emphasize that glycans are active regulators rather than passive modifiers. Importantly, they also highlight the vulnerability of the nervous system to glycosylation defects and the need for context-specific analyses. As analytical technologies, glycoengineering tools, and integrative omics approaches continue to advance, neuroglycobiology is poised to move from descriptive studies toward mechanistic and translational insights. We anticipate that future work will firmly establish glycans as central players in molecular neuroscience and promising targets for therapeutic intervention.
